# Design and *In Vitro* Characterization of Buccoadhesive Drug Delivery System of Insulin

**DOI:** 10.4103/0250-474X.40333

**Published:** 2008

**Authors:** J. Sahni, S. Raj, F. J. Ahmad, R. K. Khar

**Affiliations:** Department of Pharmaceutics, Faculty of Pharmacy, Jamia Hamdard, Hamdard University, New Delhi - 110062, India

**Keywords:** Transmucosal, buccoadhesive, protein delivery, insulin delivery

## Abstract

A buccoadhesive drug delivery system of Insulin was prepared by solvent casting technique and characterized *in vitro* by surface pH, bioadhesive strength, drug release and skin permeation studies. Sodium carboxymethylcellulose-DVP was chosen as the controlled release matrix polymer. The optimized formulation J_4_ contained Sodium carboxy methyl cellulose-DVP 2% (w/v), insulin (50 IU/film), propylene glycol (0.25 ml) and Isopropyl alcohol: water (1:4) as solvent system. Bioadhesive strength of the prepared patches was measured on a modified physical balance using bovine cheek pouch as the model membrane. *In vitro* release studies were carried out at 37 ± 2° using phosphate buffer pH 6.6, in a modified dissolution apparatus fabricated for the purpose. Cumulative amount of drug released from the optimized formulation J_4_ was 91.64% in 6 hours. *In vitro* permeation studies were carried out on J_4_ at 37 ± 2° using Franz diffusion cell. Cumulative amount of drug permeated from J_4_ was 6.63% in 6 hours. In order to enhance the permeation of protein drug, different permeation enhancers were evaluated. The results suggested that sodium deoxycholate 5% (w/v) was the best permeation enhancer among those evaluated. It enhanced the permeation of insulin from 6.63% to 10.38% over a period of 6 hours. The optimized patches were also satisfactory in terms of surface pH and bioadhesive strength. It can also be easily concluded that the system is a success as compared to the conventional formulations with respect to invasiveness, requirement of trained persons for administration and most importantly, the first pass metabolism.

Drug delivery by the transmucosal routes has gained significant attention over the last decade, particularly for the delivery of therapeutic proteins and peptides because the oral bioavailability of these drugs is usually negligible due to their poor absorption, enzymatic degradation and extensive first pass metabolism and there is a need for alternatives to the conventional parenteral route (injections) for administering them. The non-parenteral transmucosal routes commonly used include buccal^1^, sublingual^2^, rectal^3^, nasal^4^ and vaginal^5^. While each of these routes of drug administration has its associated advantages and disadvantages, the buccal route has its unique benefits. Lower enzymatic activity of saliva, easy removal of formulation, better patient acceptance and compliance are some of the prominent features of buccal route^6^–^8^.

Buccoadhesive drug delivery systems have gained considerable interest with regard to systemic delivery of drugs especially proteins and peptides which undergo extensive hepatic first pass metabolism, thus resulting in premature drug degradation within the gut^6^. Examples of some proteins and peptides delivered by buccal route include thyrotropin releasing hormone^9^, calcitonin^10^, buserdin^11^ and oxytocin^12^.

In the present study an attempt was made to formulate controlled release buccoadhesive patch for the systemic delivery of insulin. Insulin was chosen as the drug candidate, in order to overcome the limitations associated with the conventional parenteral route of the drug. The drug delivery system so found would circumvent hepatic first pass metabolism of the drug, provide painless administration of the therapeutic peptide and thereby would enhance patient compliance and acceptance. This in turn would lead to an improvement in the overall therapy of diabetes mellitus, a condition, where insulin is the most common drug of choice. Sodium carboxymethylcellulose-DVP (SCMC-DVP) is chosen as a bioadhesive and controlled release matrix-forming polymer.

One of the problems confronting the mucosal delivery of proteins and peptides e.g. insulin, is their low bioavailability, which may be either due to their metabolism at the absorption site or may be because of their poor membrane permeability. In the present study, we have tried to overcome the problem of low bioavailability of insulin, associated with its poor membrane permeability, by incorporating permeation enhancer in the formulation. Experiments were conducted to evaluate the effect of permeability enhancers i.e. β-cyclodextrin, sodium lauryl sulphate, sodium glycocholate, sodium deoxycholate, sodium laurate and glyceryl monolaurate on insulin permeability through the buccal mucosa.

## MATERIALS AND METHODS

Human insulin (100 IU/ml) was obtained from courtesy Eli Lilly, Delhi. SCMC-DVP was procured from Ranbaxy Laboratory Ltd, Delhi. Isopropyl alcohol, dichloromethane, water for HPLC, acetonitrile and methanol were purchased from E-Merck, Mumbai. Sodium dihydrogen orthophosphate, sodium hydroxide, hydrochloric acid, β-cyclodextrin, sodium lauryl sulphate, sodium glycocholate, sodium deoxycholate, sodium laurate, glyceryl monolaurate, anhydrous sodium sulphite, orthophosphoric acid and ethanolamine were purchased from S. D. Fine Chemicals Ltd., Mumbai. All solvents were of analytical/HPLC grade.

### Development of drug loaded buccoadhesive patch:

The buccoadhesive patches were prepared using solvent casting teachnique^13^. Siliconised glass plates, specially fabricated for the purpose, covered with inverted glass funnels were used as the casting assembly. The composition of the different buccoadhesive formulations is shown in Table 1.

**TABLE 1:** COMPOSITION OF INSULIN MUCOADHESIVE PATCHES

Films/Components	J_1_	J_2_	J_3_	J_4_
SCMC-DVP (%w/v)	1	1.5	2	2
Insulin (IU/patch)	10	10	10	50
Isopropyl alcohol:	(1:4)	(1:4)	(1:4)	(1:4)
Water (20 ml)				
Propylene glycol (ml)	0.25	0.25	0.25	0.25

In order to get clear polymeric solutions, free from lumps, the contents of the formulations were continuously stirred on a magnetic stirrer for over 6 h. The solutions were then poured into siliconised glass plates and solvent was allowed to evaporate at ambient conditions (temperature 37±2° and RH 45%) for 24 h. The dried films could be easily retrieved from the casting assembly and were cut into patches with a circular metallic die of 14 mm internal diameter. The patches were stuck to a backing layer made of aluminized polyester with polypropylene by using a adhesive. The buccal patches were then stored in air-tight containers at ambient conditions prior to use. Further, formulation J_4_ containing the actual dose of the therapeutic peptide (50 IU/patch) was prepared, keeping all the other ingredients same.

### Physical evaluation of mucoadhesive buccal patches:

For evaluation of film weight three patches (14 mm) of each formulation were taken and weighed individually in digital balance (Mettler-Toledo). The average weights were calculated. Three patches of each formulation were taken and the thickness was measured using Micrometer Screw Guage.

### Determination of surface pH:

For determination of surface pH three patches (14 mm) of each formulation were kept in contact with 1 ml of distilled water for 2 h, in especially fabricated glass tubes. Excess of water from the tubes was drained and the pH was noted by bringing the electrodes near the surface of the formulation and allowing it to equilibrate for 1 min^14^. A mean of three readings was recorded.

### Folding endurance:

Three patches of each formulation of size (2 × 2 cm) were cut by using sharp blade. Folding endurance was determined by repeatedly folding a small strip of the patch at the same place till it broke. The number of times the patch could be folded at the same place without breaking gave the value of folding endurance. A mean of three readings was recorded.

### Drug content uniformity:

Three patches of each formulation were taken and completely dissolved in 10 ml of pH 6.6 phosphate buffer. 2.5 μl of 9.6 N HCl was added per ml of the sample and allowed to stand for fifteen minutes. Precipitates formed were filtered out through whattman filter paper and finally membrane filtered (0.45 micron). The samples so obtained were analyzed by HPLC (Shimadzu, Japan) at 214 nm as per the procedure mentioned in USP 24.

### Measurement of bioadhesive strength of the patches:

Bioadhesive strength of the prepared patches was measured on a modified physical balance^14^,^15^. Bovine cheek pouch was used as the model membrane and isotonic phosphate buffer (IPB) pH 6.6 was used as the moistening fluid^16^. The mucosal membrane was excised by removing the underlying tissues. It was washed thoroughly with IPB pH6.6 and then tied over the protrusion in the Teflon block using a thread. The block was lowered into the glass container filled with IPB pH 6.6 at 37±2° such that the buffer just touched the sides of the mucosal membrane.

The two sides of the balance were made equal, before the study, by keeping 5.0 g weight on the right pan. The glass container was kept below the left hand side of the balance. The patch (15 mm) was stuck onto the lower side of the hanging Teflon cylinder using either a little moisture or a double sided tape.

The surface of the mucosal membrane was blotted with a whattman filter paper and 25 μl of IPB pH 6.6 was added to the mucosal surface. This was done in order to obtain reproducible results. The 5.0 g weight from the right pan was removed. This lowered the Teflon cylinder along the patch over the membrane with a weight of 5.0 g. This was kept undisturbed for 3.0 min. Then the weights on the right hand side were slowly added in increments of 0.5 g till the film just separated from the membrane surface. The excess weight on the right pan, that is, total weight minus 5.0 g was taken as a measure of the bioadhesive strength.

### *In vitro* drug release studies:

A modified dissolution apparatus, consisting of a jacketed vertical glass beaker 18 cm long and 48 cm in diameter was used for assessment of the release of drug from patches. The dissolution medium was 100 ml of IPB at pH 6.6. The patch to be evaluated was stuck on to the depression (15 mm internal diameter and 1.5mm depth) on a Teflon block fabricated for the purpose and was put into the glass beaker containing the dissolution medium. The apparatus was equilibrated to 37±2° and operated at 50 rpm.

Samples (5 ml aliquots) were pipetted out at regular intervals of time and 2.5 μl of 9.6 N HCl was added per ml of the sample and allowed to stand for fifteen minutes. Precipitates formed were filtered out through whattman filter paper and finally membrane filtered (0.45 micron). The samples so obtained were analyzed by HPLC (Shimadzu, Japan) at 214 nm as per the procedure mentioned in USP 24 using 25 cm × 4.5 mm, L1 packing (5 μm) Rp-18 Column and phosphate buffer pH 2.3:acetonitrile (74:26) as the mobile phase. Samples (5 ml aliquots) withdrawn were always replaced with an equivalent volume of IPB pH 6.6.

### *In vitro* skin permeation studies:

Formulation J_5_, J_6_, J_7_, J_8_, J_9_ and J_10_ were prepared (Table 2). A fabricated franz diffusion cell with a internal diameter of 15 mm and a diffusion area of 1.76 cm^2^ was used. The studies were performed using bovine cheek pouch. The membrane was stabilized before mounting in order to remove the soluble components present.

**TABLE 2:** COMPOSITION OF INSULIN MUCOADHESIVE PATCHES WITH DIFFERENT PENETRATION ENHANCERS

Films/Components	J_5_	J_6_	J_7_	J_8_	J_9_	J_10_
SCMC-DVP (%w/v)	2	2	2	2	2	2
Insulin (IU/patch)	50	50	50	50	50	50
Sodium lauryl sulphate (%w/v)	5	–	–	–	–	–
β-cyclodextrin (%w/v)	–	5	–	–	–	–
Sodium glycocholate (%w/v)	–	–	5	–	–	–
Sodium deoxycholate (%w/v)	–	–	–	5	–	–
Sodium laurate (%w/v)	–	–	–	–	5	–
Glyceryl monolaurate (%w/v)	–	–	–	–	–	5
Propylene Glycol (ml)	0.25	0.25	0.25	0.25	0.25	0.25

The patch was intimately attached to the upper surface of the stabilized membrane (attached b/w donor and receptor compartment), with the help of IPB pH 6.6 and a weight of 10 g for 30 s. The receptor compartment of the diffusion cell was filled with 40 ml IPB pH 7.4 and stirred at 500 rpm on a magnetic stirrer. The contents of the receptor compartment were kept at 37±2° with prewarmed water flowing through a jacket lining the receptor compartment.

The amount of drug permeated was determined by removing samples (5 ml aliquots), from the receptor compartment using a micro syringe at appropriate time intervals upto 6 h followed by their HPLC analysis. The volume withdrawn was replenished with an equal quantity of pre-warmed receptor solution. The samples were analysed by HPLC (Shimadzu, Japan) according to the procedure mentioned in USP-24.

## RESULTS AND DISCUSSION

The buccoadhesive drug delivery system of Insulin was prepared and characterized *in vitro* by physical parameters, folding endurance, content uniformity, surface pH, bioadhesive strength, drug release and skin permeation studies.

**Fig. 1:**
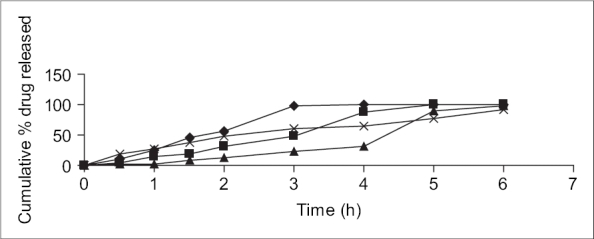
*In vitro* drug release profiles of different formulations. *In vitro* drug release from formulations J_1_ (–●–),J_2_(–■–),J_3_(–▲–) and J_4_(–×–).

The optimized mucoadhesive patch J_8_ had a thickness of 0.4±0.22 mm, weight of 110±2.16 mg and folding endurance of 211.67±2.8. The surface pH of all the patches was in the range of 5.5-7. The optimized formulation J_3_ had a satisfactory surface pH of 6.23±.09 and bioadhesive strength of 63.5±0.5. The cumulative amount of drug released in 6 h was maximum (98.18%) from formulation J_3_, containing SCMC-DVP (2% w/v) (fig. 1). From the *in vitro* drug release studies, it could also be concluded that the sustained release of insulin from the prepared formulation was directly dependent on the concentration of SCMC-DVP used.

Formulation J_3_ was chosen for further studies. The actual dose of insulin (50 IU/patch) was successfully incorporated into the patch (all the other ingredients remaining same) and the resultant formulation J_4_ was chosen for further studies. It exhibited a maximum cumulative *in vitro* release of 91.64% and maximum permeation of 6.63% in 6 h.

**Fig. 2:**
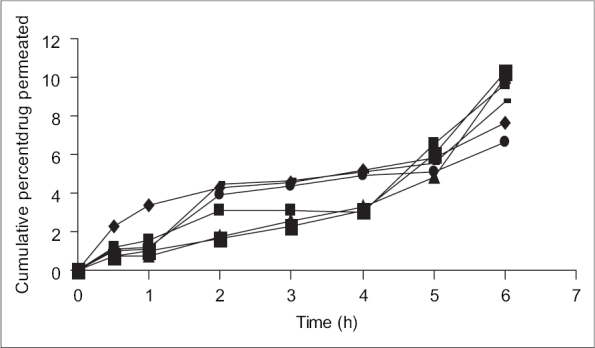
*In vitro* permeation profile of different formulations. *In vitro* permeation profiles of formulations J_5_ (–▯–), J_6_ (–■–), J_7_ (–▲–), J_8_(–▯–), J_9_ (–╺–) and J_10_ (–●–)

Permeation studies were carried on formulations J_5_, J_6_, J_7_, J_8_, J_9_ and J_10_ (fig. 2). The cumulative amount of drug permeated was maximum (10.38%) in 6 h for formulation J_8_ which contained 5% (w/v) sodium deoxycholate. Sodium deoxycholate 5% (w/v) was thus selected as the best permeation enhancer among those evaluated. It is thought to enhance the permeation of the peptide drug by solubilization of membrane components, in particular membrane proteins and lipids. Formulation J_8_ was chosen as the optimized formulation. It exhibited a maximum cumulative drug release of 91.64% and a maximum permeation of 10.38% in 6 h.

The permeation rate profile for the optimized formulation J_8_ was further analyzed for release order. A plot of logarithm of the drug remaining and time yielded a straight line, indicating a 1^st^ order release with a release constant of 0.1502 h^−1^.

In conclusion, buccoadhesive patches containing insulin were developed to a satisfactory level in terms of surface pH, bioadhesive strength, drug release and permeation. About 91.64% of the loaded drug was released *in vitro* from the drug delivery system, over a period of 6 h. Only 6.63% of the loaded insulin permeated across the bovine buccal mucosa over a period of 6 h. With the use of 5% (w/v) sodium deoxycholate, the percentage permeated increased from 6.63% to 10.38%. Analysis of the *in vitro* permeation data revealed that insulin permeated across the mucosa following a first order release pattern with a release rate constant of 0.01502 h^−1^.

It can be also be easily concluded that the system is a success as compared to the conventional formulations with respect to invasiveness, requirement of trained persons for administration and most importantly, the first pass metabolism.
